# Evaluation of reference sample type for somatic variant calling in myeloid cancers

**DOI:** 10.1007/s00277-025-06699-y

**Published:** 2025-10-22

**Authors:** Maja Z. Jakobsen, Emil-August Torp, Issa I. Issa, Helle Høholt, Mads Sønderkaer, Jakob Madsen, Lisa-Maj Christensen, Maren P. Jørgensen, Anne K. Nøhr, Rasmus F. Brøndum, Daniel T. Kristensen, Karen Dybkaer, Marianne T. Severinsen, Hanne Due

**Affiliations:** 1 Department of Hematology, Aalborg University Hospital, Søndre Skovvej 15, Aalborg, 9000 Denmark; 2 Department of Clinical Medicine, Aalborg University, Aalborg, Denmark; 3 Center for Clinical Data Science (CLINDA), Department of Clinical Medicine, Aalborg University & Research, Education and Innovation, Aalborg University Hospital, Aalborg, Denmark; 4 Biotech Research and Innovation Centre (BRIC), Faculty of Health and Medical Sciences, University of Copenhagen, Copenhagen, Denmark; 5 Department of Hematology, Rigshospitalet, Copenhagen University Hospital, Copenhagen, Denmark; 6 Department of Molecular Diagnostics, Aalborg University Hospital, Aalborg, Denmark; 7 Department of Hematology, Aarhus University Hospital, Aarhus, Denmark; 8 Department of Molecular Medicine, Aarhus University Hospital, Aarhus, Denmark; 9 Department of Clinical Medicine, Aarhus University, Aarhus, Denmark; 10 Clinical Cancer Research Center, Aalborg University Hospital, Aalborg, Denmark

**Keywords:** Myeloid cancers, Somatic variant calling, Whole exome sequencing, Clinical hematology, Precision medicine

## Abstract

High-throughput sequencing is transforming cancer diagnostics, research, and clinical decision-making, yet accurate identification of disease-driving somatic variants often requires comparing tumor and normal DNA. This study aimed to identify the optimal normal reference sample for whole exome sequencing (WES) in myeloid-derived cancers. T-cells, skin biopsies, saliva, and a tumor-only analysis were evaluated by comparing variant calls from tumor-reference pair to those from tumor-fibroblast analyses. T-cells demonstrated the highest sensitivity (0.91-1.00) and lowest false positive rate, establishing them as a reliable reference option. Skin biopsies and saliva yielded inconsistent results, likely due to leucocytic contamination. While tumor-only analysis offered high sensitivity, it generated numerous false positives; however, a selective filtration strategy provides a feasible solution by omitting the need for a normal reference sample. These findings suggest T-cells are the optimal balance of accuracy and practicality for identifying somatic variants in myeloid cancers, while tumor-only analysis presents a viable alternative with careful filtering. This improved workflow supports efficient and reliable genomic profiling for personalized treatment decisions. ClinicalTrials.gov, TRN: NCT05695638, Registration date: 10 January 2023.

**Supplementary Information:**

The online version contains supplementary material available at 10.1007/s00277-025-06699-y.

## Dear editor-in-chief

Advances in high throughput sequencing have led to novel insights into the genetic landscape of cancer, shifting the primary challenge from technical sequencing technicalities to data interpretation and applying it for diagnosis, prognosis, and treatment guidance. For myeloid derived cancers, genetic profiles have provided critical new information on disease etiology, risk stratification models, and prognostic and predictive markers [1]. Personalized medicine initiatives have further emphasized the significance and place of sequencing in a clinical setting [2].

As most genetic variants in cancer are somatic, a reference normal sample is often included to enable individualized subtraction of germline variants from tumor sequencing data to identify only somatically acquired genetic variants [1]. Nevertheless, differentiating somatic from germline variants are challenged by the presence of innocuous polymorphisms and age-related accumulation of clonal hematopoiesis-associated variants [3].

Since myeloid neoplasms arise from genetic alterations in early myeloid progenitor cells, somatic mutations may be detectable in multiple tissues containing myeloid derived cells throughout the body [4]. This introduces the risk of neoplastic contamination in reference samples, potentially leading to the failure to detect somatic variants.

The focal point of this study was to identify optimal reference sample type for calling somatically acquired variants from whole exome sequencing (WES) in myeloid derived cancers. To this end, variant counts from four potential normal sample types were compared to those from patient-derived fibroblasts. While fibroblast samples are considered the gold standard for confirming diagnoses of hereditary hematological malignancies using sequencing [5, 6], this approach is significantly limited by a slow and labor-intensive workflow, which impedes its widespread clinical application. To our knowledge, this is the first study to evaluate the performance of multiple reference sample types for variant calling in next-generation sequencing by using fibroblast as benchmark [7–9].

Eleven patients with myeloid derived cancers confirmed by the WHO criteria [4] were enrolled in the ProSeq Cancer trial (NCT05695638/N-202000118) at Aalborg University Hospital, Denmark. The ProSeq Cancer is a regional initiative to provide a personalized treatment approach based on WES to relapsed or refractory patients with advanced and incurable cancer [10]. Informed written consent was obtained in accordance with the Declaration of Helsinki [11]. Four patients were excluded from the current study because of allogenic stem cell transplantation prior to sampling (*n* = 2), failure to culture fibroblasts (*n* = 1), and sequencing challenges (*n* = 1). Fibroblast and T-cell collected from bone marrow aspirate in addition to saliva and skin biopsy were sampled at relapse and examined as reference sample types, and variants from each of these were evaluated relative to fibroblast. In addition, a tumor-only bioinformatic analysis was included to assess its potential for elucidating somatic variants, potentially obsoleting the use of a reference tissue. DNA from nail clippings were also explored as a prospective reference tissue in a few pilot tests, but was excluded as the material was prone to degradation and did not meet quality criteria for WES.

Of the seven patients for which data are presented, diagnoses were as follows: acute myeloid leukemia (*n* = 1); chronic myeloid leukemia (*n* = 1); myelodysplastic syndrome (*n* = 2); myelodysplastic syndrome/myeloproliferative neoplasm with neutrophilia (*n* = 2); mixed phenotype acute leukemia (*n* = 1). The cohort included six males and one female, and the median age at time of inclusion was 58 (range 22–80). Tumor and reference samples; fibroblast (*n* = 7), T-cell (*n* = 5), skin biopsy (*n* = 7), and saliva (*n* = 5) were subjected to Illumina paired-end sequencing (Supplementary Material), and data were processed using an in-house developed bioinformatic workflow [2, 12]. Further information on sample collection (Supplementary Fig. S1) and processing is available in Supplementary Material.

The mean coverage for WES, in the regions considered for analysis, was 344 (range 237–466) for tumor samples, and 189 for reference samples (180 for fibroblast [range 115–225], 178 for saliva [range 149–214], 181 for skin biopsy [range 84–256], and 261 for T-cell [range 181–352], Table 1). Variants were considered as true positives when called in tumor-fibroblast analyses, which yielded a median of 23 true positive somatic variants (range 6–45). Performance of variant calling using each reference sample type was evaluated by comparing them to tumor-fibroblast to assess sensitivity, the presence of false positives, and missing variants. The latter constitute variants undetected in tumor-only, skin biopsy, T-cell, and saliva, respectively.

**Table 1:** Summary data for whole exome sequencing of reference and tumor-only samples

	Fibroblast			Tumor-only			T-cell			Skin biopsy			Saliva		
P	**Variants**	**VAF**	**Depth**	**Variants**	**VAF**	**Depth**	**Variants**	**VAF**	**Depth**	**Variants**	**VAF**	**Depth**	**Variants**	**VAF**	**Depth**
1	20	0.43	323	19	0.43	463	-	-	-	13	0.42	56	-	-	-
	0	-	-	314	0.57	357	-	-	-	4	0.18	25	-	-	-
2	31	0.14	293	30	0.14	365	29	0.15	203	29	0.15	214	-	-	-
	0	-	-	297	0.53	265	2	0.10	18	4	0.10	60	-	-	-
3	45	0.30	263	44	0.29	397	44	0.30	312	18	0.32	373	45	0.30	266
	0	-	-	311	0.59	293	1	0.35	9	0	-	-	0	-	-
4	6	0.40	379	6	0.40	405	6	0.40	459	6	0.40	261	6	0.40	350
	0	-	-	275	0.55	279	0	-	-	2	0.09	76	1	0.21	22
5	31	0.35	246	29	0.37	572	-	-	-	0	-	-	1	0.11	393
	0	-	-	366	0.54	382	-	-	-	4	0.10	140	1	0.08	41
6	23	0.41	145	23	0.41	509	21	0.43	366	16	0.44	287	1	0.05	115
	0	-	-	329	0.57	403.58	0	-	-	0	-	-	0	-	-
7	24	0.38	147	22	0.37	290	22	0.40	219	5	0.33	113	1	0.07	39
	0	-	-	305	0.55	297	0	-	-	2	0.08	43	2	0.10	39

Overview of variants for reference sample types and tumor-only analysis for the patient 1–7. True and false positive variant metrics annotated in blue and red, respectively. Variants reported met QC criteria of allele frequency > 0.05 and at least 5 reads supporting the alternative allele. *P* patient, *VAF* variant allele frequency noted as detected in the associated tumor sample and reported as average values

**Fig. 1:**
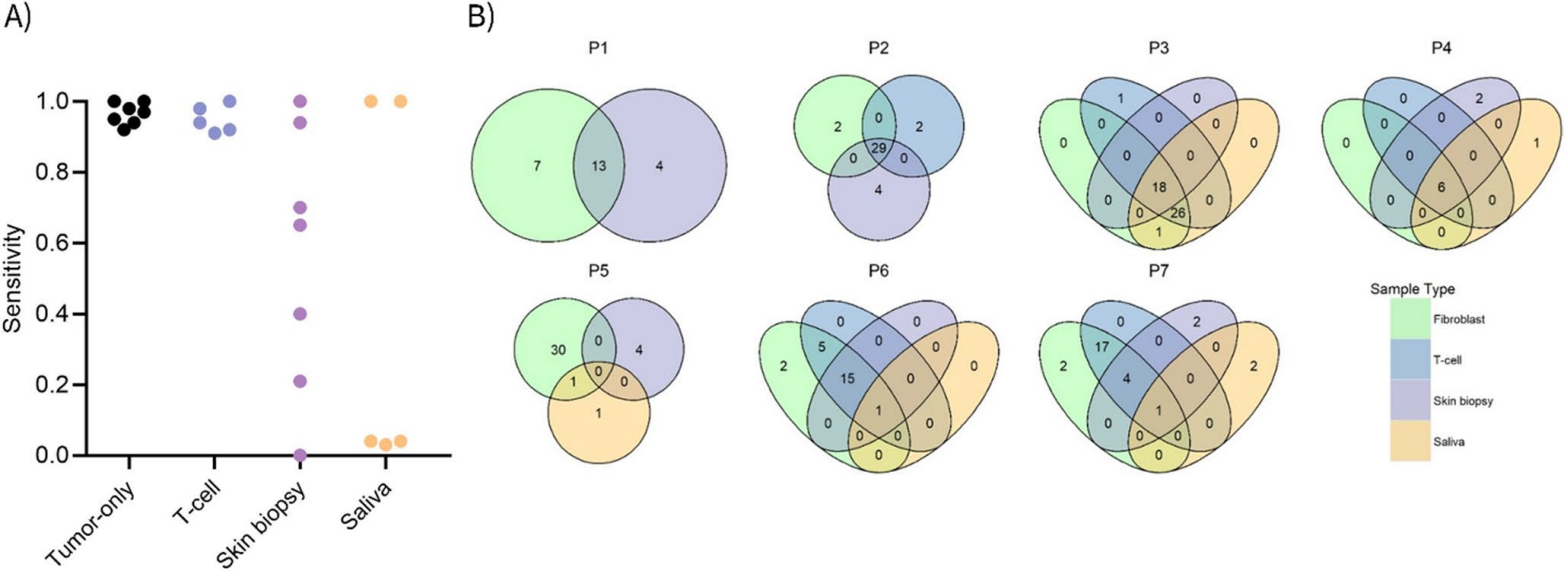
(**A**) Sensitivity plot reflecting the capability of the respective reference sample types to call positive variants, i.e., somatic variants called in tumor-fibroblast. Each dot represents a patient. Data was visualized using graphpad prism v.10.1.1. (**B**) All variants detected in tumor-fibroblast compared to t-cell, skin biopsy, and/or saliva for patient 1–7 visualized in venn diagrams. Tumor-only was excluded due to the high number of false positive variants

The highest sensitivity was observed for tumor-only (range 0.92–1.00) and T-cell (range 0.91–1.00), where the former yielded the highest number of true positive variants (Fig. 1A, Supplementary Table S2). A distinct variation in sensitivity was observed between patients when calling variants using skin biopsies and saliva as reference sample. While skin biopsies depicted evenly distributed sensitivity scores ranging from 0.00 to 1.00, performance for saliva samples were confined to the extremes with three cases performing poorly reflecting 22–30 missing variants per sample (P5, P6, and P7, Supplementary Table S2) while two cases reached a sensitivity of 1.00 (Fig. 1A, Supplementary Table S2), suggestive of a perfect concordance between observed and expected true positive variants. The elevated rate of missing variants in both saliva and skin biopsy samples is likely a consequence of neoplastic contamination from leucocytes either already present in the sample or introduced during sample acquisition.

To evaluate true and false positives for individual patients, variants identified from each tumor-reference pair were compared to variants called from tumor-fibroblast analyses (Table 1) and visualized in Venn diagrams displaying the overlap in called somatic variants between reference samples (Fig. 1B), except for tumor-only as addressed in the following.

In general, a substantially higher number of false positive variants were called when using the tumor-only analysis (Table 1) with a median of 309 false positives (range 275–366) contributing to the highest number of false positives detected per true positive variants with a median of 14 (Supplementary Table S2). Of note, the high number of false positives called from the tumor-only analysis is most likely derived from calling of germline variants since an average VAF of 0.54–0.59 was observed across patient tumor samples. In contrast to the high number of false positives observed for the tumor-only pipeline, the average number of false positives (ratio between false- and true positives provided in parenthesis) was 1.5 (0.02), 3.2 (0.17), and 1.3 (0.63) for T-cell, skin biopsy, and saliva, respectively (Table 1, Supplementary Table S2, Fig. 1B).

Lastly, in two patients, we observed three variants that were missed when using T cells as the reference but correctly detected in the tumor-only analysis, potentially suggesting overlap in contributions from myeloid and T-cell lineages. Manual IGV inspection revealed low-level variant-supporting reads in the T-cell samples; however, none of the loci mapped to known clonal hematopoiesis-associated genes [13], leaving their origin unresolved. Similar low-frequency signals were observed at bona fide somatic loci, but the markedly lower VAFs in T cell relative to tumor ensured correct somatic classification.

In clinical practice, sequencing supports clinical decision-making, and a high degree of sensitivity is pertinent. The frequencies in interpersonal variation from skin biopsies and saliva samples imply that these sample types are inadequate as normal reference because of the large number of missing variants, presumably as a result of tumor contamination [7, 8]. The extent of tumor DNA contamination in skin or saliva samples is likely dependent on disease burden at the time of sampling and thus may be less pronounced in patients tested at remission compared to those at initial diagnosis or relapse. Owing to the large number of false positives, tumor-only analysis requires stringent filtration steps or an assigned list of known tumor genes and oncogenes of clinical interest, which is feasible in a routine clinical setting, especially since reference samples can be omitted. However, this approach might introduce other challenges in research settings due to the laborious individual filtration steps required, which can be difficult to automate.

While utilizing patient-derived fibroblasts as a reference sample may reduce the likelihood of missing variants [14], the time required for fibroblast culturing is incompatible with the rapid diagnostic need for treatment initiation. A limitation of using T cell as a germline comparator is that clonal hematopoiesis–associated variants, which originate in hematopoietic progenitors, can occasionally be detected in both myeloid and lymphoid lineages [15] and shared variants could theoretically obscure detection of corresponding tumor variants. In our study, we observed 3 missed variants all with low tumor VAFs. While using T-cell as reference sample has this potential, albeit rare, drawback, one approach to mitigate this issue might be setting a VAF ratio between tumor and normal samples to augment the sensitivity of the bioinformatical pipeline. Based on this study, we recommend using T-cell as the reference sample when using next-generation sequencing to identify somatic variants in patients with myeloid-derived cancers in line with previous findings [8, 9]. This recommendation is supported by the high sensitivity and low number of false-positives observed with T-cells, as well as the feasibility in T-cell isolation, which can be readily performed simultaneously with flow cytometry for myeloid tumor cell isolation from bone marrow aspirates.

## Supplementary Information

Below is the link to the electronic supplementary material.

Supplementary Material 1 (DOCX 643 KB)

## Data Availability

The data generated and analyzed during the current study are not publicly available due to the sensitive nature of the data involving personal information. Data access is restricted in accordance with legal and ethical data protection policies. Researchers may contact the corresponding author for information about potential data access under appropriate data-sharing agreements and ethical approvals.
