# Supplementary material for: Evaluation of reference sample type for somatic variant calling in myeloid cancers
Source: Ann Hematol. 2025 Oct 22;104(11):6091–5. doi: 10.1007/s00277-025-06699-y (PMC12672828; doi:10.1007/s00277-025-06699-y)
Supplement: Supplementary file 1 — Supplementary Material 1 (DOCX 643 KB) [file 277_2025_6699_MOESM1_ESM.docx]

**Supplementary Material**

*Collection of biological material*

Bone marrow (BM) aspirates and routine peripheral blood (PB) samples were obtained at diagnosis or relapse, and mononuclear cells (MNCs) were isolated by Ficoll separation (GE Healthcare) and processed directly or cryopreserved until further processing.

Saliva samples were collected in ORAGENE-DNA collection tubes (DNAgenotek, Cat. No. OG-600), and 4 mm skin biopsies were obtained using a 2 mm biopsy punch (Hounisen, cat. No. 0017.0082), placed in RNA*later*™ (Invitrogen, Cat. No. AM7020) and stored for 1-4 days at 5 °C until processing. Initially, nail was assessed as potential reference source but exhibited poor DNA yield and was consequentially discarded.

*Sample preparation for WES*

Fibroblast monocultures were established from patient-specific BM-MNC isolates by culturing in media consisting of DMEM (Gibco, cat. No 21969-035) supplemented with 10% fetal calf serum (Gibco, cat. No 105000-064), 1% PenStrep (Thermo Fisher, cat. No. 15140122), 2mM L-Glutamine (Sigma Aldrich, cat. No. G7513), and 111 µM 2-Mercaptoethanol (Sigma Aldrich, cat. No. M3148) and split twice weekly. Depending on cell density, fibroblasts were harvested after ~ 3 weeks in culture, and cell lineage was validated by immunophenotyping using flowcytometry.

Malignant myeloid cells and T-cells from BM-MNCs were isolated and enriched by Fluorescence-activated cell sorting (FACS) on a SH800S Cell Sorter (Sony Biotechnology, Japan) using a multiplex panel of fluorescently tagged fluorochromes (Supplementary Fig. S1). Cell populations of interest were sorted based on manual gating strategies to select cells based on 1) size and granularity ensuring exclusion of cellular debris and doublets; 2a) myeloid tumor cells were defined CD45^+^ and CD34^-^ and CD117^-^, and 2b) T-cells were isolated based on CD3^+^, CD45^+^, and CD14^-^ and CD19^-^. Fibroblast cell lineage was confirmed by positive expression of CD90 and CD105 and negative expression of CD33, CD34 and CD45. Overview of immunophenotyping markers applied for sorting is listed in Supplementary Table S1, and example of gating strategy provided in Supplementary Fig. S2. All staining procedures were carried out as recommended by distributor. FACS of malignant myeloid cells was omitted for patients harboring a blast count of > 65% as determined by the Department of pathology, Aalborg University Hospital in concordance with procedures for standard diagnostic pipeline.

DNA from saliva was purified using the prepIT™-L2P kit (DNAgenotek, Cat. No. PT-L2P-5), while DNeasy® Blood & Tissue Kit (Qiagen, Cat. No., 69504) was utilized for DNA isolation of skin biopsies and MNCs from FACS, both in accordance to the manufacturers’ protocols in addition to the user-Developed Protocol “*Purification of total DNA from soft tissues using the TissueLyser and the DNeasy® Blood & Tissue Kit*”for skin biopsies.

DNA quality and quantity was assessed using Qubit (Invitrogen, Carlsbad, CA, USA) and Nanodrop (Thermo Scientific, Waltham, MA, USA), and 200 ng of DNA was used as input for WES.

*WES*

WES library preparation was performed as previously described [1]. Target minimum average coverage of 75x and 150x was chosen for normal and tumor WES samples, respectively. Paired-end sequencing was performed on a NextSeq 500 or a NovaSeq 6000 (Illumina, San Diego, CA, USA) yielding a minimum of 26 Gb reads for tumor DNA and 18 Gb reads for reference DNA.

*Bioinformatic processing*

An in-house workflow, previously described in [1], was adjusted to current versions software versions, and used for the samples in this project.

Reads were aligned to the GRCh38.d1.vd1 reference genome using BWA mem v0.7.17 for WES reference and tumor samples. Somatic variant detection was performed using Mutect2 [2] v4.2.6.1 and filtered using VCFtools v0.1.13 to keep variants with a "PASS" filter label within coding regions +10bp, based on refseq genes (Annotation Release110). The resulting VCF files were annotated using GATK Funcotator [2] version 4.6.1.0 with Funcotator data source v1.8.hg38.20230908s and saved as MAF files.

Further analysis was done in R v4.4.2 [3] using vcfR v1.15.0 [4] and maftools v2.22.0 [5] to process the VCF and MAF files, respectively. Variants with VAF>0.05 and at least 5 reads supporting the alternative allele were used for analysis of sensitivity to ensure high quality data. Variants meeting QC criteria encompassed single nucleotide variants and variants with median length of 3 bp (range 1-180 bp).

Unique IDs were assigned to the variants in the form of [Chromosome:Position:Reference:Alternative] and used to identify concordant variants across samples for different reference sample types and calculate sensitivity (Supplementary Table S2):

$$Sensitivity = \frac{TP}{TP+FN}$$

To evaluate the risk of false positive variant calling in the tumor-only and reference samples, a ratio between false positive variants and true positive variants (Supplementary Table S2) was calculated by:

$$FP ratio = \frac{FP}{TP}$$

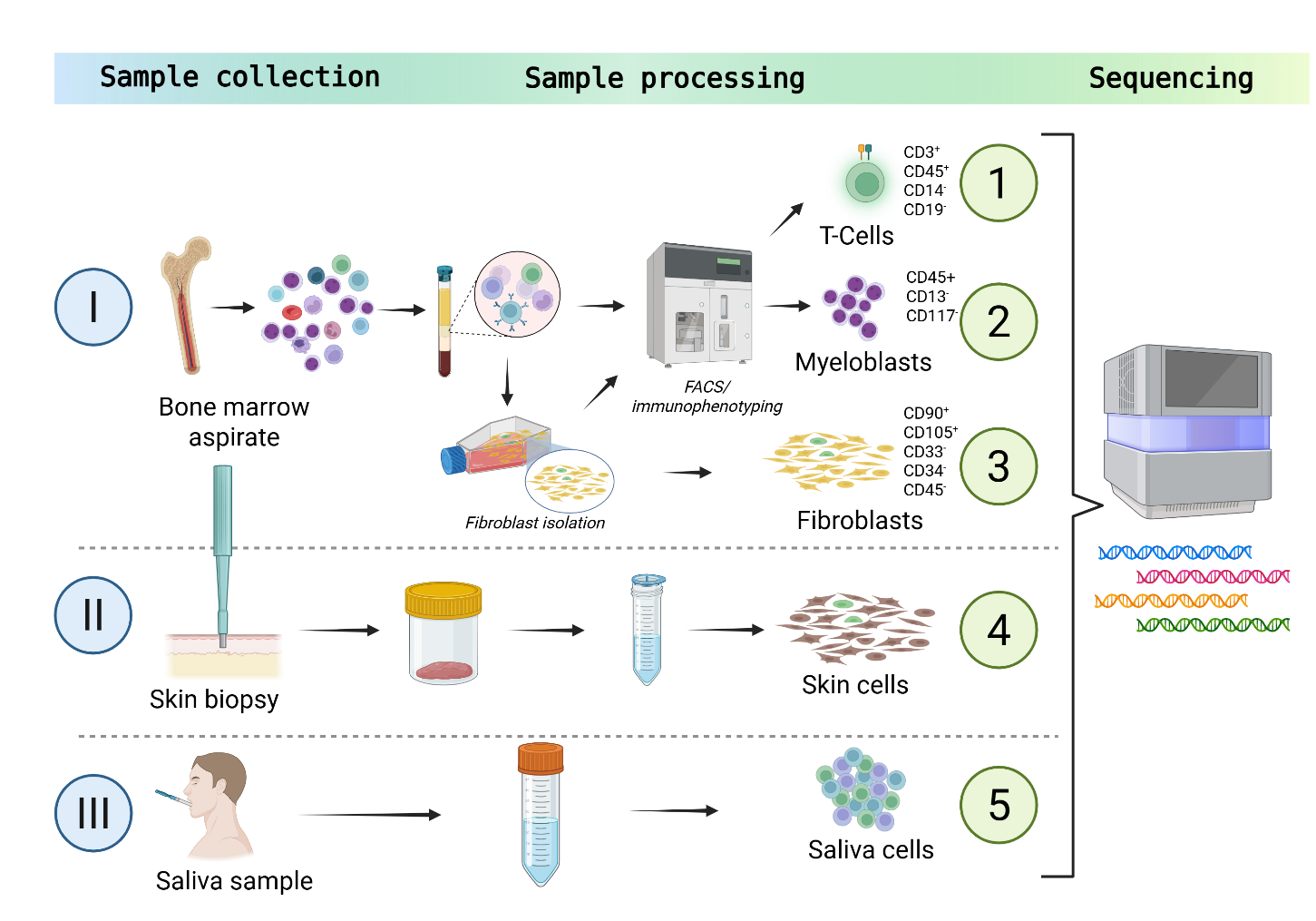


**Supplementary Fig. S1**. Overview of workflow for isolation of tumor and the four reference samples. Created with BioRender.com

| Supplementary Table S1 | | | |
| --- | --- | --- | --- |
| *Surface marker* | ***Fluorochrome*** | ***Distributor*** | ***Cat. No.*** |
| CD3 | FITC | DAKO | F0818 |
| CD14 | APC | Becton Dickens | 34787 |
| CD19 | PE-Cy7 | Beckman Coulter | IM3628 |
| CD33 | APC | Becton Dickens | P67 |
| CD34 | PerCP-Cy5.5 | Becton Dickens | 347222 |
| CD45 | Pacific Blue | DAKO | PB986 |
| CD90 | FITC | BD Biosciences | 555595 |
| CD105 | PE | BD Biosciences | 560839 |
| CD117 | APC | DAKO | C724401-2 |

*
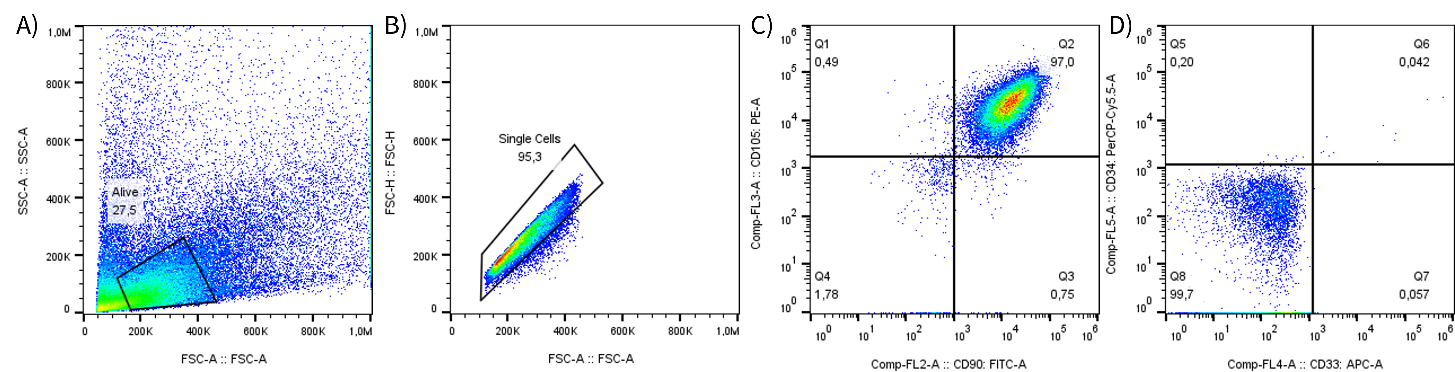
*

**Supplementary Fig. S2**. Manual gating strategy for immunophenotyping and FACS. A) Determination of live fibroblast population by forward and side scatter followed by doublet discrimination (B). CD90 and CD105 positive cells (C) were confirmed to be devoid of CD33, CD34 (D), and CD45 (not shown) expression. Density plots were generated using FlowJo v.10.10.0.

| Supplementary Table S2 | | | | | | | | |
| --- | --- | --- | --- | --- | --- | --- | --- | --- |
|  | ***Tumor-only*** | | ***T-cell*** | | ***Skin biopsy*** | | ***Saliva*** | |
| Patient | **Sensitivity** | **FP/TP ratio** | **Sensitivity** | **FP/TP ratio** | **Sensitivity** | **FP/TP**  **ratio** | **Sensitivity** | **FP/TP ratio** |
| 1 | 0.95 | 16.5 | - | - | 0.65 | 0.3 | - | - |
| 2 | 0.97 | 9.9 | 0.94 | 0.07 | 0.94 | 0.1 | - | - |
| 3 | 0.98 | 7.1 | 0.98 | 0.02 | 0.40 | 0 | 1.00 | 0 |
| 4 | 1.00 | 45.8 | 1.00 | 0 | 1.00 | 0.3 | 1.00 | 0.2 |
| 5 | 0.94 | 12.6 | - | - | 0.00 | 4 (Inf) | 0.03 | 1 |
| 6 | 1.00 | 14.3 | 0.91 | 0 | 0.70 | 0 | 0.04 | 0 |
| 7 | 0.92 | 13.9 | 0.92 | 0 | 0.21 | 0.4 | 0.04 | 2 |
| True positive and false positive variants called in tumor-only analysis or in tumor-reference pair (when available, otherwise denoted by -) by comparing to tumor-fibroblast analysis were leveraged to assign sensitivity and false positive (FP)/true positive (TP) ratio for patient 1-7. Inf: Infinum | | | | | | | | |

References

[1] J. S. Bødker *et al.*, “Development of a precision medicine workflow in hematological cancers, Aalborg university hospital, Denmark,” *Cancers (Basel)*, vol. 12, no. 2, p. 312, Jan. 2020, doi: 10.3390/cancers12020312.

[2] D. Benjamin, T. Sato, K. Cibulskis, G. Getz, C. Stewart, and L. Lichtenstein, “Calling Somatic SNVs and Indels with Mutect2,” Dec. 02, 2019. doi: 10.1101/861054.

[3] The R Core Team, “A Language and Environment for Statistical Computing,” Vienna, Jun. 2025. Accessed: Jun. 20, 2025. [Online]. Available: https://www.R-project.org/

[4] B. J. Knaus and N. J. Grünwald, “VcfR: a package to manipulate and visualize variant call format data in R,” *Mol Ecol Resour*, vol. 17, no. 1, pp. 44–53, Jan. 2017, doi: 10.1111/1755-0998.12549.

[5] A. Mayakonda, D. C. Lin, Y. Assenov, C. Plass, and H. P. Koeffler, “Maftools: Efficient and comprehensive analysis of somatic variants in cancer,” *Genome Res*, vol. 28, no. 11, pp. 1747–1756, Nov. 2018, doi: 10.1101/gr.239244.118.
